# Therapeutic potential of conditioned medium obtained from deferoxamine preconditioned umbilical cord mesenchymal stem cells on diabetic nephropathy model

**DOI:** 10.1186/s13287-022-03121-6

**Published:** 2022-09-02

**Authors:** Serbay Ozkan, Basak Isildar, Merve Ercin, Selda Gezginci-Oktayoglu, Dildar Konukoglu, Neşet Neşetoğlu, Mahmut Oncul, Meral Koyuturk

**Affiliations:** 1 Histology and Embryology Department, Cerrahpasa Faculty of Medicine, Istanbul University-Cerrahpasa, Kocamustafapaşa Street, 34098 Istanbul, Turkey; 2 Biology Department, Molecular Biology Section, Faculty of Science, Istanbul University, Istanbul, Turkey; 3 Medical Biochemistry Department, Cerrahpasa Faculty of Medicine, Istanbul University-Cerrahpasa, Istanbul, Turkey; 4 Faculty of Pharmacy, Drug Application and Research Center, Istanbul University, Istanbul, Turkey; 5 Cerrahpasa Faculty of Medicine, Obstetrics and Gynecology Department, Istanbul University-Cerrahpasa, Istanbul, Turkey

**Keywords:** Deferoxamine, Diabetic nephropathy, Mesenchymal stem cell, Preconditioning, Conditioned medium

## Abstract

**Background:**

The therapeutic potential of mesenchymal stem cells (MSCs)-derived conditioned media (CM) can be increased after preconditioning with various chemical agents. The aim of this study is comparative evaluation of effects of N-CM and DFS-CM which are collected from normal (N) and deferoxamine (DFS) preconditioned umbilical cord-derived MSCs on rat diabetic nephropathy (DN) model.

**Methods:**

After incubation of the MSCs in serum-free medium with/without 150 µM DFS for 48 h, the contents of N-CM and DFS-CM were analyzed by enzyme-linked immunosorbent assay. Diabetes (D) was induced by single dose of 55 mg/kg streptozotocin. Therapeutic effects of CMs were evaluated by biochemical, physical, histopathological and immunohistochemical analysis.

**Results:**

The concentrations of vascular endothelial growth factor alpha, nerve growth factor and glial-derived neurotrophic factor in DFS-CM increased, while one of brain-derived neurotrophic factor decreased in comparison with N-CM. The creatinine clearance rate increased significantly in both treatment groups, while the improvement in albumin/creatinine ratio and renal mass index values were only significant for D + DFS-CM group. Light and electron microscopic deteriorations and loss of podocytes-specific nephrin and Wilms tumor-1 (WT-1) expressions were significantly restored in both treatment groups. Tubular beclin-1 expression was significantly increased for DN group, but it decreased in both treatment groups. Terminal deoxynucleotidyl transferase dUTP nick end labeling (TUNEL)-positive apoptotic cell death increased in the tubules of D group, while it was only significantly decreased for D + DFS-CM group.

**Conclusions:**

DFS-CM can be more effective in the treatment of DN by reducing podocyte damage and tubular apoptotic cell death and regulating autophagic activity with its more concentrated secretome content than N-CM.

**Graphical Abstract:**

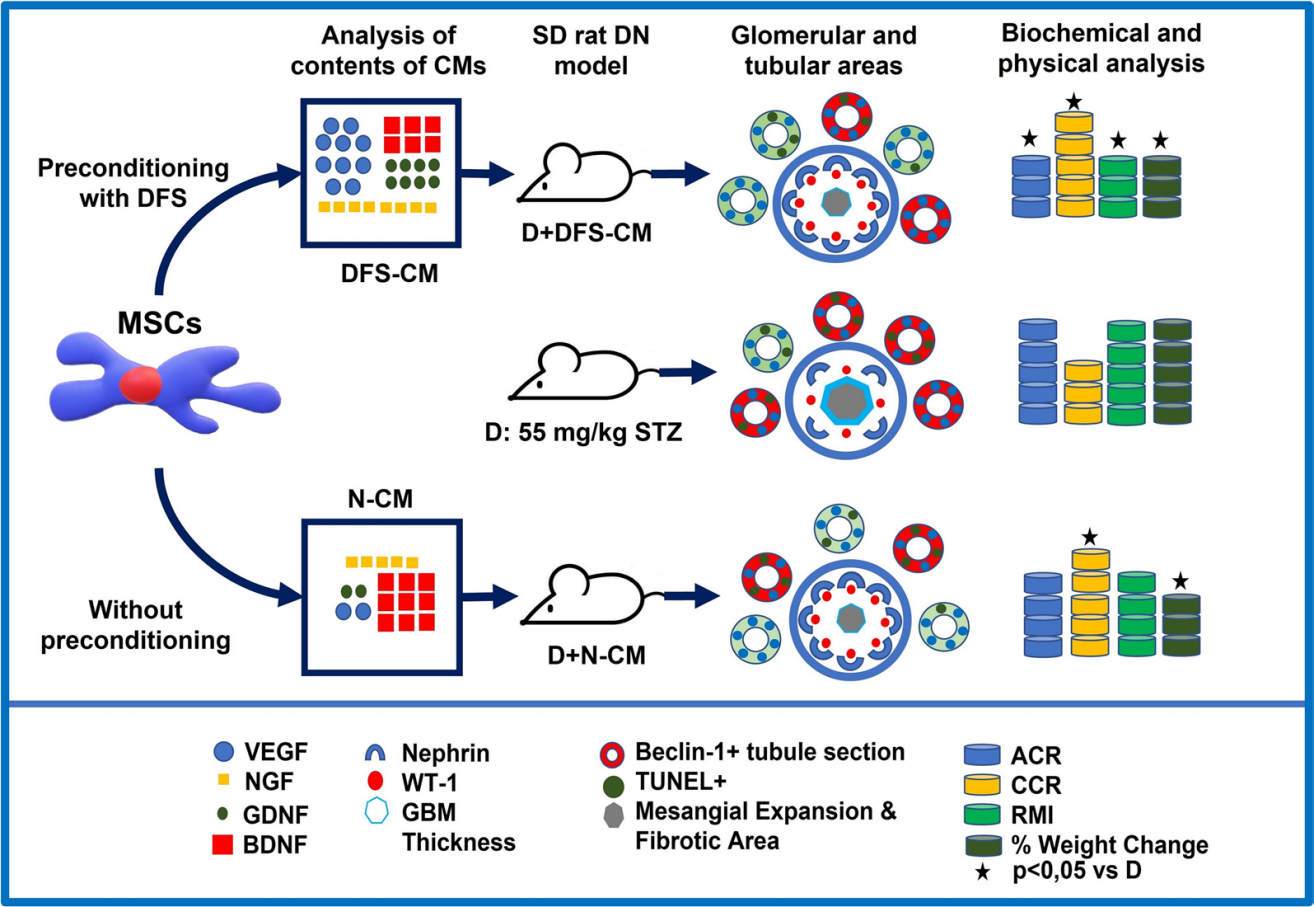

**Supplementary Information:**

The online version contains supplementary material available at 10.1186/s13287-022-03121-6.

## Background

Diabetic nephropathy (DN) which is a microvascular complication of diabetes mellitus (DM) is the most common cause of chronic kidney disease. Approximately one-third of DM patients develop DN, and it was estimated that DN will be the seventh most common cause of death by 2030 [1, 2]. The pathogenesis and pathophysiology of renal diseases like DN is primarily associated with glomerular damage of which focus point is podocyte injury and loss. In association with podocyte damage, albuminuria, glomerular capillary dysfunction, thickening of glomerular basal membrane (GBM), expansion of mesangial area, glomerular hypertrophy and fibrosis in advanced stages have been noted for the pathology of DN [3, 4]. The pathogenesis of glomerular damage with respect to podocytes has been associated with hypertrophy which is considered as an adaptation for the covering of naked GBM surface resulting from glomerular dilation, epithelial–mesenchymal transition, apoptosis and dysregulated autophagy [3, 5, 6]. On the other hand, it has been claimed that tubular regions are the primary site of injury in against to general paradigm of which pathogenesis of renal diseases is based on the glomerular area [7]. The study with human urinary sample bio-markers showed that proximal tubule damage is prominent in the early period of DN progression without the occurrence of glomerular damage [8, 9]. High rate of reabsorption which accounts for the 60% to 70% of the ultrafiltrate, accordingly being exposed to hyperglycemia and advanced glycation end products (AGEs) and development of local hypoxia are inducing factors in the mechanism of DN-related proximal tubule injuries [7, 10]. Those findings indicate that tubules specifically proximal ones could be the primary injury site in the progression of renal function loss independent of glomerular damages [11, 12]. It has been shown that apoptotic cell death in renal epithelial cells increases due to hyperglycemia [13, 14]. In addition, dysregulation of the autophagic mechanism plays an important role in the development of DM-related renal tubule damage [15, 16]. However it was noted that the role of autophagic mechanism could differ in the different kidney injury models [17, 18].

Even if applications of drugs including anti-hypertension drugs or sodium glucose co-transporter 2 protein inhibitors can slow down the progression of the disease, they are not able to prevent the disease from progressing to end-stage kidney disease [19–21]. Mesenchymal stem cells (MSCs) have come up as potentially therapeutic for the treatment of broad range of diseases including DN [1, 22]. The recent studies showed that direct application of MSCs has therapeutic effects even though they are not localized to target tissue but to the other organs primarily to the lungs which is the situation indicates that MSCs show their therapeutic effects mainly through the paracrine interactions [23, 24]. Conditioned medium (CM) obtained from the MSCs includes various kinds of soluble factors including growth factors, cytokines, chemokines and non-soluble extracellular vesicles all of which are defined as secretome [25, 26]. It was shown that direct application of MSCs or CMs obtained from MSCs has similar level of therapeutic effects in the treatments of various kinds of in vivo disease models [25, 26]. Furthermore, the CMs have important advantages facilitating their therapeutic use over MSCs such as easier transporting and storing and not requiring cryopreservation [27].

It was shown that stemness of MSCs isolated from patients with diabetes or metabolic syndrome was significantly reduced [28, 29]. In this respect, the recent studies showed that preconditioning of MSCs with chemicals like lipopolysaccharides and interferon-gamma at under the lethal dose could improve the therapeutic potentials of CMs obtained from those cells [30, 31]. Deferoxamine (DFS) which is used in the treatment of iron and aluminum toxicities has hypoxia mimetic effects. Adipose tissue-derived MSCs treated with DFS have increased hypoxia-inducible factor-1alpha (HIF-1α) expression [30]. Furthermore, MSCs treated with DFS have increased secretion of VEGF-α and augmented expressions of neuro-protective growth factors like nerve growth factor (NGF), glial cell-derived neurotrophic factor (GDNF) and brain-derived neurotrophic factor (BDNF) at mRNA level [30]. Neurons and podocytes have a lot of common characteristic features. Both of them are terminally differentiated and have common characteristic protein expressions like nephrin, synaptopodin, similar cytoskeletal organization and cellular interaction processes [32–34]. At this point, it was separately shown that neuro-protective growth factors including NGF, GDNF and BDNF have important roles for podocytes during the differentiation, apoptotic resistance and protection of cytoskeleton organization [35–37]. Moreover, pro-angiogenic VEGF-α plays an important role in the protection of glomerular capillary endothelial function and fenestrated structure [12]. Therefore, when the protective effects of growth factors on podocytes and the microvascular nature of DN are considered, utilizing neuron-podocyte similarity, and applying pro-angiogenic factors would be effective strategy for the treatment of DN. So that modifying the content of CM obtained from MSCs in favor of neuro-protective and pro-angiogenic growth factor could improve the therapeutic effects of normal CM on DN. The aim of this study was to compare the growth factor contents of two types of CMs collected after incubation of human umbilical cord-derived MSCs in normal and DFS preconditioning serum-free culture mediums and their therapeutic effects on the rat DN model.

## Material and method

### Isolation and characterization of UC-MSCs

MSCs were isolated from the human umbilical cord (UC) on the basis of their ability to adhere to plastic surface [38, 39]. UC was obtained from a pregnant woman who had no known disease giving birth by caesarean section at full term and immediately transported to the laboratory in Leibovitz medium (Pan-Biotech: P04-27,500) supplemented with 2% of antibiotic antimycotic solution under cooled condition and MSCs were isolated by tissue explant method as described previously [38, 39]. When 3rd generation (P3) of adherent cells was obtained, characterizations of the cells were performed. Morphologies were evaluated with invert microscopy. Immunophenotypes of the cells were analyzed by the assessment of expression levels of human MSC-specific CD44 (PE conjugated), CD90 (APC conjugated) and non-MSC-specific CD34 (FITC conjugated), CD45 (APC-Cy conjugated) cell surface markers (BD Biosciences) with flow cytometry as described previously [38]. Adipogenic and osteogenic differentiation potential of the cells was detected with Oil Red O and Alizarin Red S staining, respectively, after incubation with corresponding differentiation inducing mediums (Sigma, 811D-250 and Sigma, 417D-250, respectively) [40].

### Preconditioning of UC-MSCs with DFS and collection of CMs

**Fig. 1:**
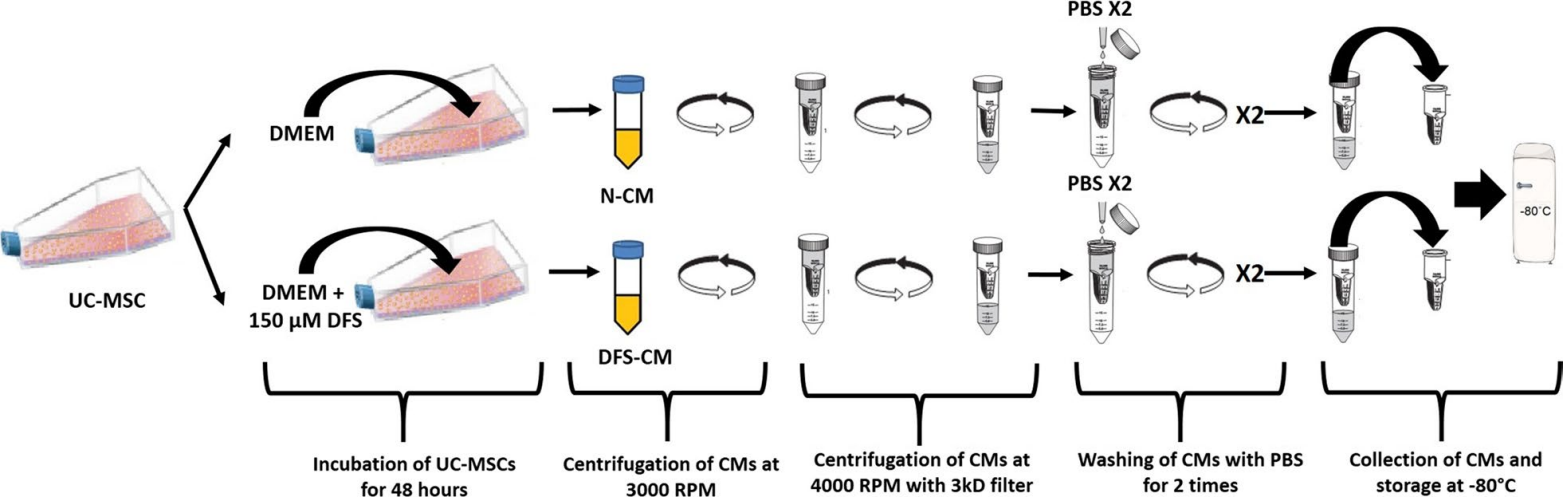
The process of obtaining conditioned mediums

UC-MSCs (3rd passage) at 70–80% confluency were incubated in 15 ml of serum-free DMEM/F12 medium (ThermoFisher, 11320074) with double-distilled water (vehicle) or 150 µM deferoxamine (DFS) for 48 h in T75 flask. At the end of the incubation period, mediums were collected as normal conditioned medium (N-CM) and DFS preconditioned conditioned medium (DFS-CM). The cells remaining on the plate were harvested, and the percentage of dead cells was determined with trypan blue exclusion assay. The collected CMs were centrifuged at 3000 rpm for 5 min to remove cell debris, and the supernatants were tenfold concentrated by centrifuging at 4000 rpm for 45 min with 3kD cutoff filters (Merck, UFC900324). Using the same filters, PBS was added to the concentrated medium and centrifugated at 4000 rpm for 45 min and then the last step was repeated to thoroughly eliminate DFS from the medium. The obtained concentrated CMs were stored at -80 ºC for further analysis and in vivo use (Fig. 1).

#### *Determination of DFS in CMs by high-performance liquid chromatography-mass spectrometry*

 The presence of DFS in the content of CMs obtained from MSCs was evaluated before washing, after the first and the second washes by high-performance liquid chromatography-mass spectrometry. For this, liquid chromatography-mass-mass spectrometry instrument (Agilent Technologies Triple Quadrupole 6460) was used. The samples were injected by using the mobile phase consisting of LC–MS grade methanol: 2% formic acid (95:05, v/v) mixture. It was analyzed for 2 min with 1.0 ml/min of mobile phase flow rate and 3 µl of injection volume. The mass/charge (m/z) ratios of the precursor ion and the product ion were determined as 561,6 and 201,2, respectively, and the analysis of those ions was performed in the mass detector.

### Bicinchoninic acid and enzyme-linked immunosorbent assays

Total protein concentration of the CMs obtained under two different conditions was determined by the bicinchoninic acid (BCA) assay kit (Abbkine: KTD3001). The concentrations of NGF (Boster EK0469), BDNF (Boster EK0307), GDNF (Boster EK0362) and VEGF-*α* (Abbkine KET6033) growth factors in the contents of CMs were determined with enzyme-linked immunosorbent assay (ELISA) by following the protocols suggested by the commercial kits.

### Immunocytochemistry analysis

HIF-1α expressions were determined with immunocytochemistry staining. For this purpose, MSCs seeded on coverslips in 24-well plates were incubated in serum-free DMEM/F12 medium with/out DFS for 48 h; then, the medium was removed. After washing with PBS, the cells were fixed with paraformaldehyde. The fixative was removed from the medium and washed with PBS. After permeabilization with 0,1% PBS-Triton X-100, blocking was performed (ScyTek Laboratories SensiTek HRP kit). Following overnight incubation with 5 µg/ml HIF-1α antibody (Boster A00013), the cells were washed with PBS and incubated with biotinylated secondary antibody (ScyTek Laboratories SensiTek HRP kit). After washing, incubation with streptavidin peroxidase solution was performed. Color development was detected with the application of 3-amino-9-ethylcarbazole (AEC) chromogen. The samples were examined with the Olympus BX61 microscope and photographed with the Olympus DP72 camera.

### Rat DN model and treatment

Sprague Dawley male rats (350–400 g; 10–12 weeks old) were used in this study, and they were kept under standard conditions defined with 12 h of dark & light cycle, 20 ± 2 ℃ temperature, 45–55% humidity and free access to water and food.

**Fig. 2:**
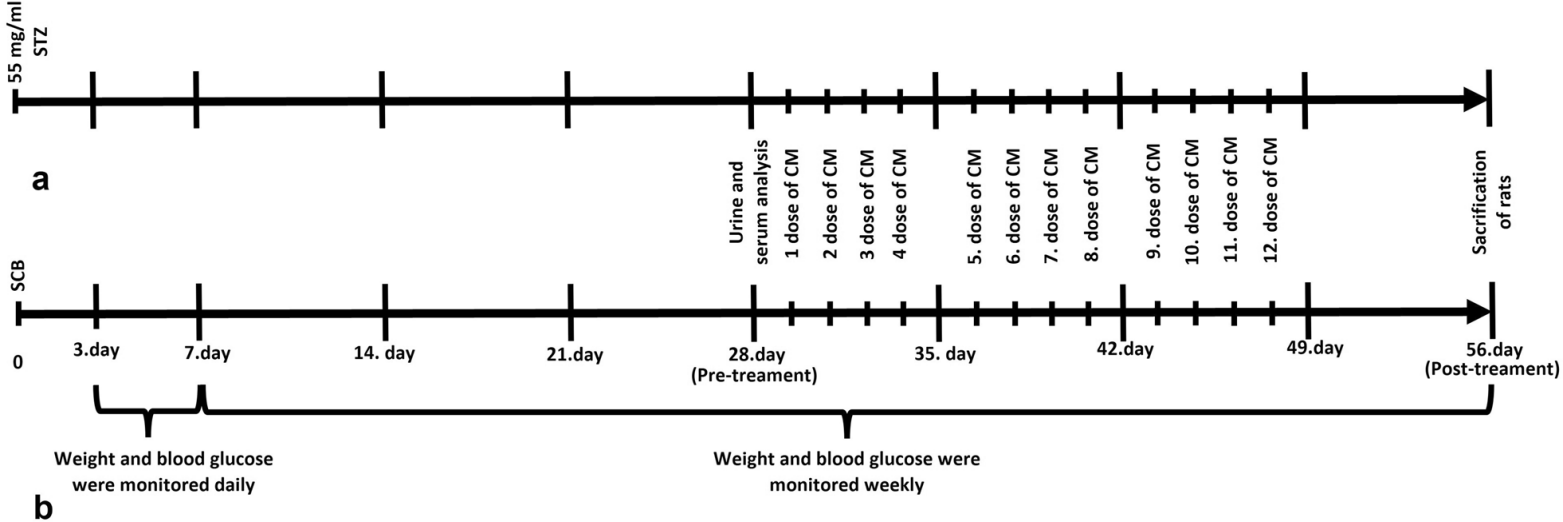
Timelines for experimental **a** and control **b** groups

All rats were randomly divided into the control group (*N* = 6) and diabetic groups (*N* = 20). Diabetes was induced by intraperitoneal administration of 55 mg/kg of single-dose streptozotocin [STZ (Sigma S0130) dissolved in sodium citrate buffer (SCB, pH: 4,5)] after 12 h of fasting, while the control rats were administrated with the equal volume of SCB as vehicle treatment. After 72 h, the blood glucose level was measured from the tail vein with a glucometer (Contour Plus, Bayer). Rats with blood glucose level greater than 250 mg/ml were considered diabetic and then followed for 1 week [41]. At the end of the 4th week following the STZ injection, the urinary albumin/creatinine ratio (ACR) was evaluated and the rats with ACR greater than 93 mg/g were diagnosed with diabetic nephropathy and were included in the experiments [29]. Twenty rats with DN were divided into 3 groups; the first group (*N* = 6) was left without any treatment, and the second (D + N-CM, *N* = 7) and the third (D + DFS-CM, *N* = 7) groups were intraperitoneally administrated with N-CM and DFS-CM, respectively. Equal volumes of two types of CMs with minimum contents of 15 µg protein in each dose were administrated at 4 doses a week for 3 weeks following the development of DN. The rats were killed under general anesthesia of ketamine and xylazine at the end of the 8th week (Fig. 2).

During the experiments, body weights and blood glucose levels were measured weekly. After killing, kidneys were weighted to calculate renal mass index [RMI = (kidney weight/body weight) × 100]. At the end of the 4th week and 8th week before the killing of the rat, urine and serum samples were collected to confirm the development of DN model and to monitor renal function, respectively. Blood samples obtained from the jugular vein and 24-h urine samples collected in metabolic cages were centrifugated at 2000 g for 10 min. Serum creatinine (Scr) and urine creatinine (Ucr) levels were determined in the Roche HITACHI Cobas c 501 module of which working principle is based on the kinetic calorimetric Jaffé method. Urine albumin levels were measured with colorimetric ELISA by following the protocol suggested by the manufacturer (ElabScience E-EL-R0362). Renal function was assessed by calculating the urinary albumin/creatinine ratio (ACR) and the creatinine clearance rate [CCR = Scr/Ucr x V (V: urine volume (ml) per minute)].

### Light microscopic analysis

The right kidneys of the rats were divided into two in the sagittal plane and prepared for the light microscopic investigations as described Additional file 1: S1. In order to evaluate the degrees of tubular damage (dilation and vacuolization), increase in interstitial area (edema or inflammatory cell infiltration), amount of cell or cellular debris in tubule lumens, expansion in mesangial area, thickening in GBM and fibrosis in the glomerular region, the section was stained with hematoxylin & eosin (H&E), periodic acid Schiff & hematoxylin (PAS&H) and Masson’s trichrome and then they were examined with Olympus BX61 microscope and photographed with an Olympus DP72 camera [42–44].

### Transmission electron microscopic (TEM) analysis

The renal cortex regions of left kidneys were carefully dissected and minced into 1 mm^3^ pieces. The samples were subjected to fixation and preparation procedure as described in Additional file 1: S2. Ultrastructural changes in the cellular compartments of glomerular and cortical tubular areas were thoroughly evaluated. Furthermore, two different morphometric analyses were performed on 5 different TEM images obtained from peripheral regions of glomerulus. First, GBM thickness was determined by measuring the vertical distance between the endothelial cell and podocyte membranes. Podocyte foot retraction or effacement was evaluated by calculating the mean width of the podocyte foot processes. For this, the number of slit diaphragms was counted along the 7000 nm length of GBM and the mean width of the foot process was calculated by dividing the GBM length by the number of slit diaphragm.

### Immunohistochemical analysis

*Nephrin and Wilms tumor-1 (WT-1)*: Following rehydration of deparaffinized sections, antigen retrieval was performed in TRIS–EDTA buffer (pH:9,0) for 20 min in the microwave. After washing with PBS, the sections were incubated in 3% H_2_O_2_ for 25 min at RT. After washing with PBS, blocking (SensiTek Super Block) was applied for 6 min. Then, the sections were separately incubated with primary antibodies of 1 µg/ml WT-1 (Abcam, ab212951) and 0,607 µg/ml nephrin (Abcam, ab216341) at + 4 ℃ overnight. Following washing with PBS, the instructions of secondary antibody kit (ScyTek Laboratories SensiTek HRP kit) were followed as it was mentioned in staining protocol. Analysis of immunohistochemical stainings was performed on an average 30 glomeruli by evaluating the nephrin staining intensity per glomerulus with the Fiji ImageJ software program and counting the WT-1-positive cells in each glomerulus.

#### Beclin-1

Deparaffinized and rehydrated sections were incubated in citrate buffer (pH:6,0) for 20 min in the microwave for antigen retrieval. After washing with tris-buffered saline (TBS), the sections were incubated in 3% H_2_O_2_ solution for 25 min at RT. Following washing with TBS, blocking was performed with 5% bovine serum albumin (BSA) prepared in PBS for 1 h. Afterward, the sections were incubated with beclin-1 (1/100 dilution; Abbkine, ABM0079) primary antibodies in 5% BSA at + 4 °C overnight. In the next steps, the instructions of secondary antibody kit (SensiTek) were followed. Analysis of immunoreactivity of beclin-1 was performed by evaluating 10 randomly selected areas from the cortical region at 200X magnification. In this respect, the numbers of beclin-1-positive cells in the glomeruli and the number of tubule sections containing beclin-1-positive cells in the tubular area were determined. Afterward, the amount of tubule sections containing beclin-1-positive cells per area and the amount of beclin-1-positive cells per glomeruli were calculated for each sample.

#### Terminal deoxynucleotidyl transferase dUTP nick end labeling (TUNEL) analysis

For TUNEL analysis, the protocol recommended by the manufacturer (Abcam ab206386) was followed. For each sample, 15 randomly selected areas from the cortical region at 200X magnification were evaluated. TUNEL-positive cell numbers in the glomerular and tubular areas were counted, and the average positive cell numbers were calculated separately.

### Statistical analysis

Statistical analyses were performed with SPSS version 21.0 statistical program. Data were expressed as mean ± standard error. The data of the groups were tested with the Shapiro–Wilk normality test for whether they are normally distributed or not. Parametric one-way analysis of variance (one-way ANOVA) was used to compare the means of the groups with normal distribution and when a significant difference was found with ANOVA; comparisons between paired groups were made with the post hoc Tukey test. In the absence of normality, the significance between the groups was evaluated with nonparametric Wilcoxon signed rank or Kruskal–Wallis tests. *P* < 0.05 was considered statistically significant [45].

## Results

### Characterization of UC-MSCs

**Fig. 3:**
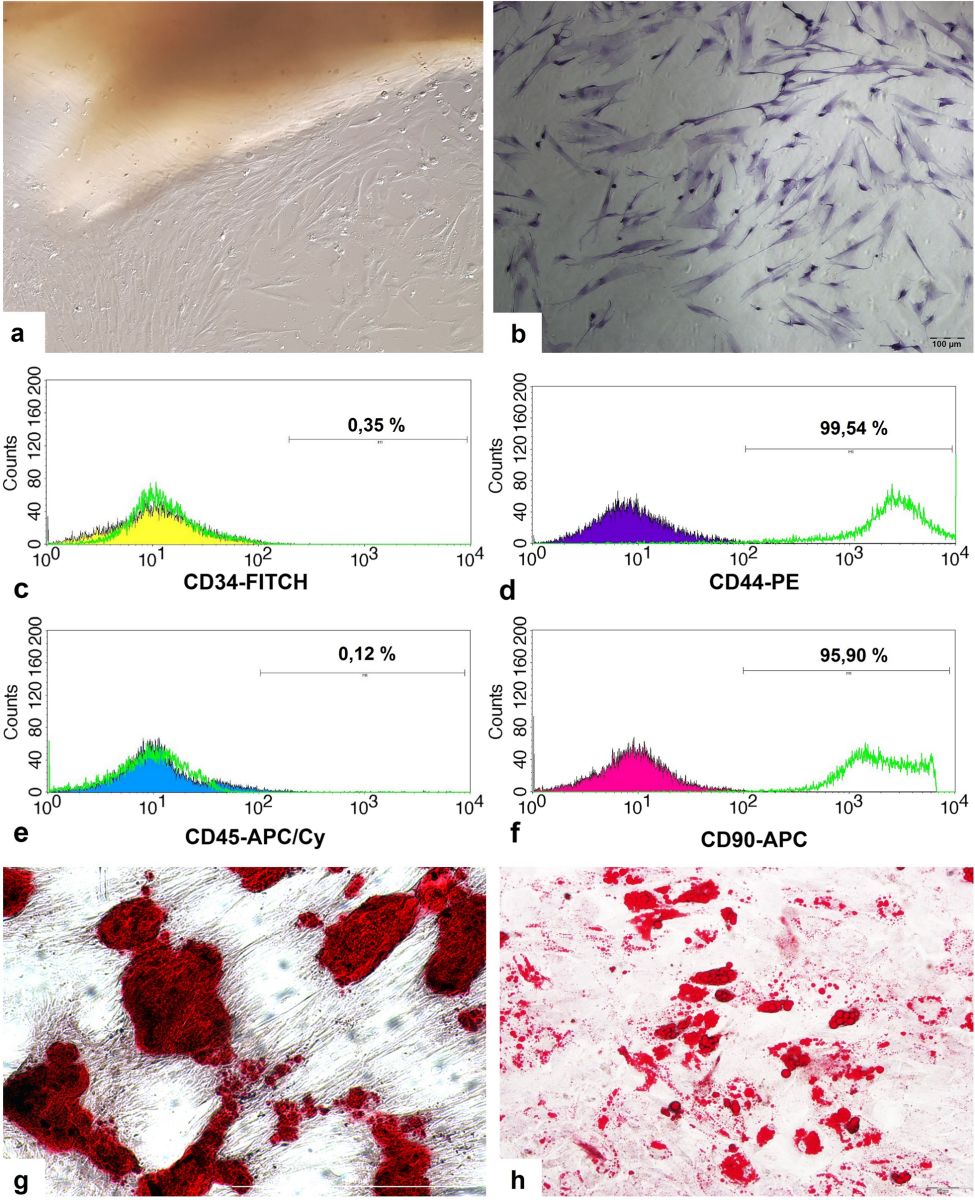
Spindle-shaped cells observed around the tissue explant (100X) **a**. Illustration of spindle or star-shaped morphology of the isolated cells by H&E staining **b**. Histogram graphics of flow cytometric analysis of cells labeled with CD34 **c**, CD44 **d**, CD45 **e** and CD90 **f** antibodies. Representative figures for osteogenic **g** and adipogenic **h** differentiation potentials of hUC-MSCs

HUC-MSCs were isolated by the tissue explant method which is based on their ability to adhere to the plastic surface. At the end of the 10th day, spindle-shaped cells were observed around the UC tissues (Fig. 3a). At the end of the 16th day, Petri dishes which have 90% confluency were harvested and 1st passage (P1) cells were obtained. The MSCs at 3rd passage had typical morphology like star or spindle cell shapes (Fig. 3b). The expression levels of MSC-specific CD44, CD90 and non-MSC-specific CD34, CD45 cell surface markers were determined as 99,54%, 95,90% and 0,35%, 0,12%, respectively, by flow cytometric analysis (Fig. 3c–f). Multipotency of the MSCs was evaluated by inducing adipogenic and osteogenic differentiation. Afterward, osteogenic and adipogenic differentiations were demonstrated by Alizarin Red S and Oil Red O two of which stain the extracellular calcium deposits and the lipid droplets in the cytoplasm of cell as red, respectively (Fig. 3g–h).

### Evaluation of effects of preconditioning on cell viability and HIF-1α expression

**Fig. 4:**
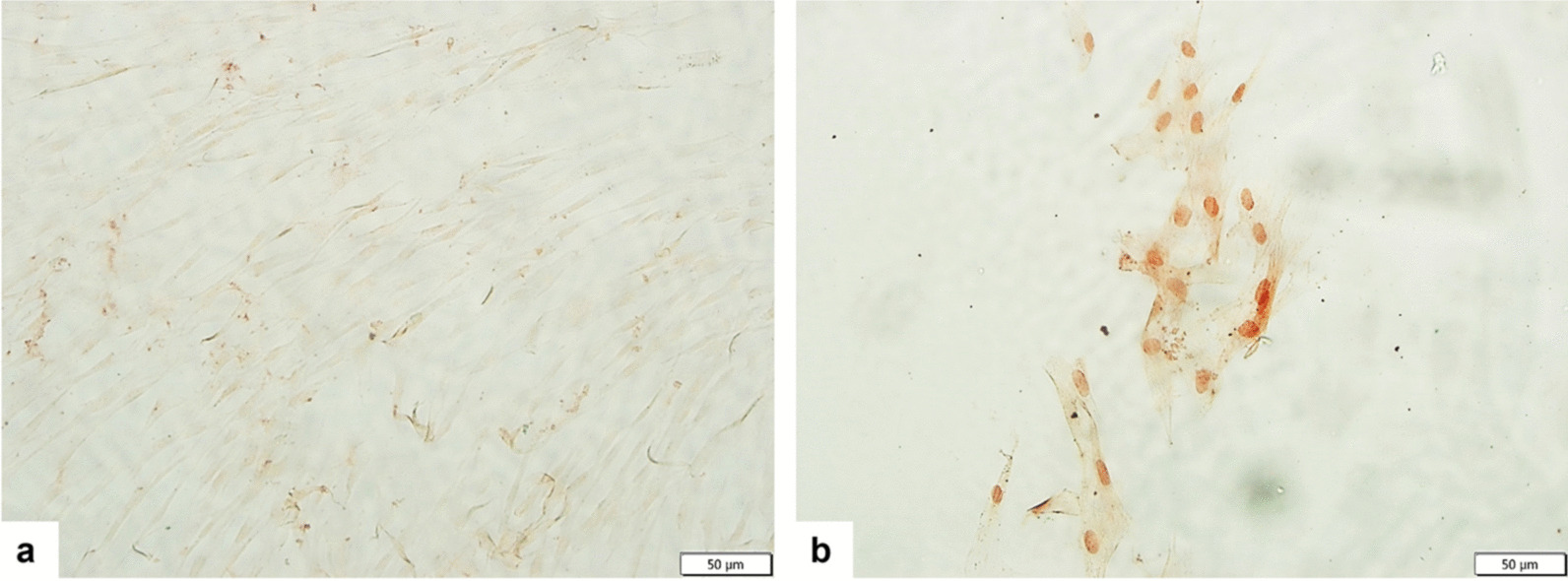
Immunocytochemical analysis of HIF-1*α* nuclear expressions in normal **a** and DFS preconditioned **b** MSCs

DFS-CM and N-CM were collected after incubation of hUC-MSCs in serum-free DMEM/F12 medium with 150 µM DFS or vehicle. The effect of DFS on cell viability was evaluated by trypan blue exclusion assay, and the cell death ratio was determined as 1,3%. HIF-1*α* expressions in the MSCs were determined with immunocytochemical staining. There was no HIF-1α expression in the MSCs incubated in normal serum-free medium, while distinctive nuclear HIF-1α expression was detected in MSCs preconditioned with 150 µM DFS for 48 h (Fig. 4).

### Quantification of concentration of total protein and growth factors in CMs

**Fig. 5:**
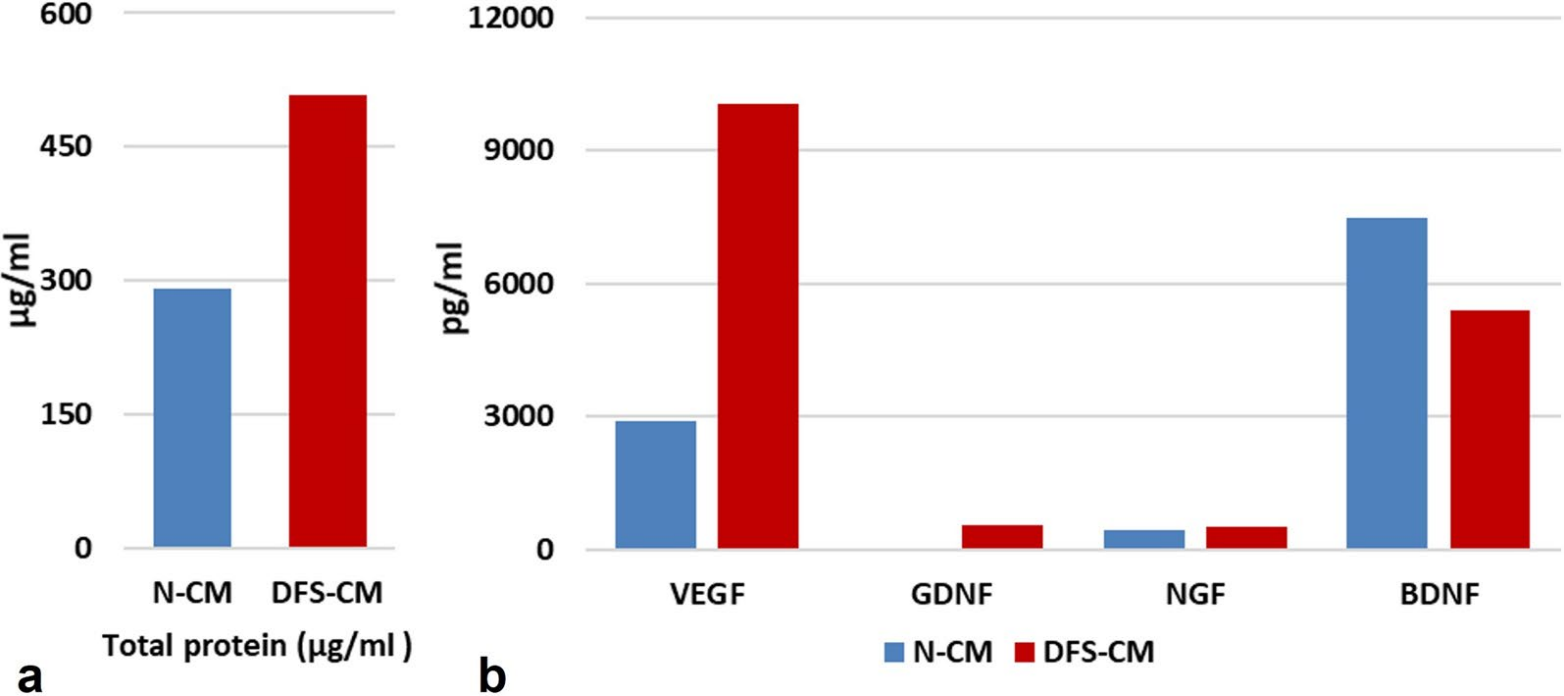
Quantitative comparisons of total protein **a** and growth factors **b** in the contents of N-CM and DFS-CM

Total protein concentrations of N-CM and DFS-CM were determined by BCA assay as 290,98 µg/ml and 507,83 µg/ml, respectively (Fig. 5a). The quantifications of VEGF-*α*, GDNF, NGF and BDNF growth factors in the secretome of CMs were performed by ELISA method. The concentrations of VEGF-*α* (2896 pg/ml and 10,064 pg/ml), GDNF (20 pg/ml and 544 pg/ml) and NGF (426 pg/ml and 517 pg/ml) in DFS-CM were found to be increased compared to N-CM (Fig. 5b). On the contrary, concentration of BDNF was found to be decreased in DFS-CM (7486 pg/ml and 5410 pg/ml) compared to N-CM (Fig. 5b).

### Determination of DFS in the CMs’ contents

Whether DFS was completely eliminated at the end of the washing processes, the samples were tested with high-performance liquid chromatography-mass spectrometry analysis. As can be seen in Additional file 2: Fig. S1, DFS was completely removed in the end of the second wash.

### Biochemical and physical analysis

**Fig. 6:**
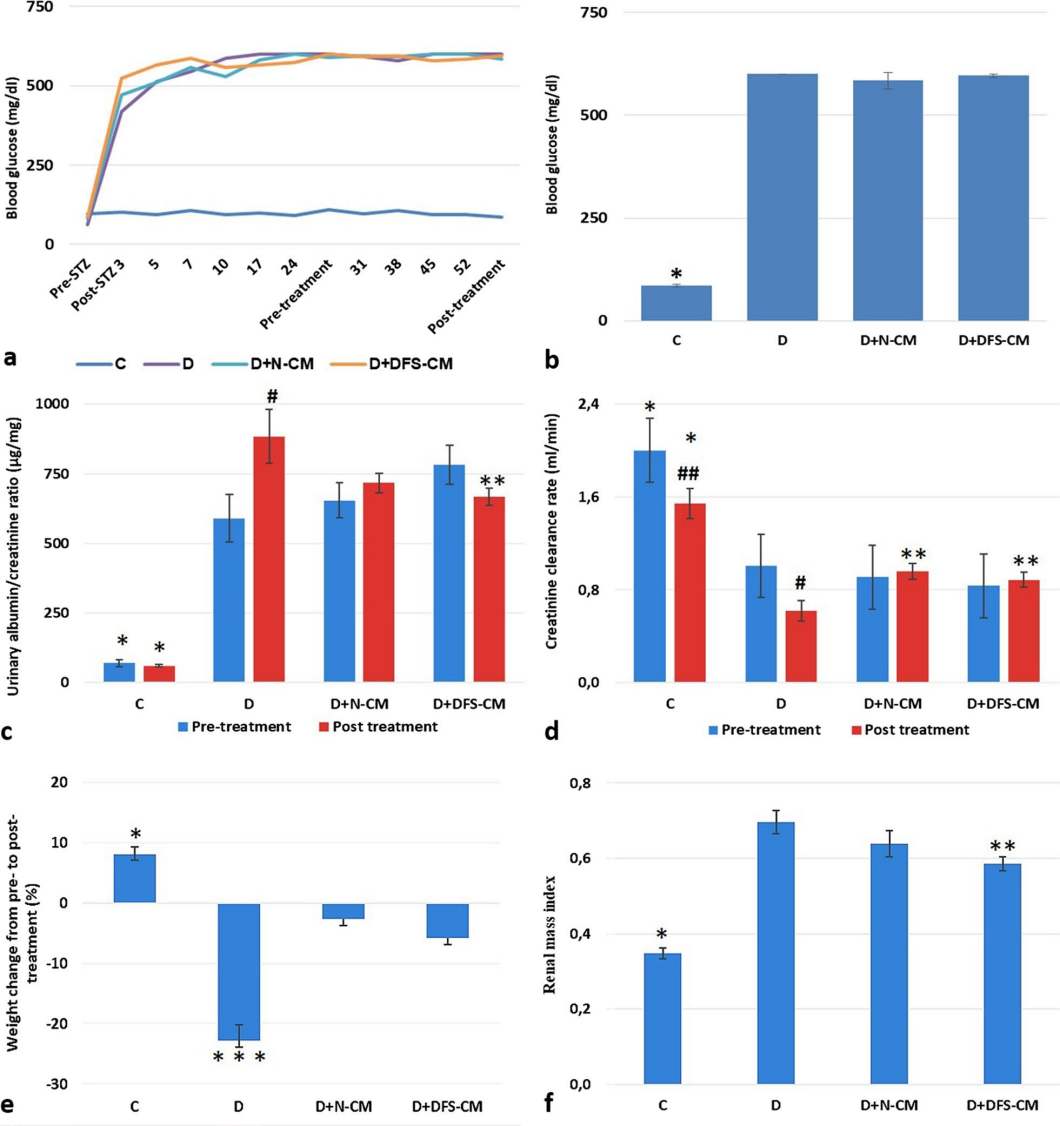
Biochemical and physical analysis of rats. Change in blood glucose level during **a** and in the end of experiments **b**. Graphical representation of urine albumin/creatinine ratio (µg/mg) **c** and creatinine clearance rate (ml/min) **d**. Percent weight change from pre- to post-treatment **e**. Rat renal mass indices **f**. *P < 0,001 vs D, D + N-CM, D + DFS-CM; **P < 0,05 vs D; ^#^*P* < 0,05 vs pre-treatment D; ^##^*P* < 0,01 vs pre-treatment C; ***P < D + N-CM, D + DFS-CM

At the 3rd day following STZ injection, the blood glucose levels of rats were measured in the diabetic range which was determined as > 250 mg/ml (Fig. 6a). In the following weeks, no significant change was observed for blood glucose levels in both the control and experimental groups (Fig. 6a–b). At the end of the 4th week following STZ injection, urinary albumin/creatinine ratios (ACRs) and creatinine clearance rates (CCRs) of diabetic and control rats were compared. ACRs values of control and diabetic rats were measured as 70 ± 12 and 679 ± 43 ug/mg, respectively, and this increase was found to be statistically significant (*p* < 0,0001) (Fig. 6c). CCRs values of diabetic rats (0,91 ± 0,05 ml/min) were found to be significantly decreased compared to control ones (2,00 ± 0,15 ml/min) (*p* < 0,0001) (Fig. 6d). These findings indicated that the DN model was successfully established at the end of the 4th week following the STZ injection. Afterward, the diabetic rats were homogeneously divided into experimental groups according to their ACR and CCR values (D, D + N-CM, D + DFS-CM). In the end of the experiment, in each one of the experimental groups the ACRs were found to be significantly increased, while CCRs were significantly decreased compared to control group (*p* < 0,001) (Fig. 6c–d). ACRs decreased in both D + N-CM (716 ± 35 ug/mg) and D + DFS-CM (666 ± 30 ug/mg) groups compared to D (884 ± 97 ug/mg) group, but the decrease was only significant for D + DFS-CM (*p* < 0,05) (Fig. 6c). Furthermore CCRs were found to be significantly increased in both D + N-CM (0,95 ± 0,06 ml/min) and D + DFS-CM (0,88 ± 0,06 ml/min) groups compared to D group (0,61 ± 0,08 ml/min) (Fig. 6d).

Finally, pre- and post-treatment values of ACR and CCR were compared for all groups separately. In the C group, there was no significant change in ACR (70 ± 12 ug/mg and 60 ± 5 ug/mg), while CCR (2,00 ± 0,15 ml/min and 1,54 ± 0,13 ml/min) was decreased significantly in post-treatment period (*p* < 0,05) (Fig. 6c–d). On the other hand, in group D, ACR (589 ± 85 ug/mg and 884 ± 97 ug/mg) increased and CCR (1,00 ± 0.09 ml/min and 0,61 ± 0,09 ml/min) decreased significantly compared to pre-treatment period (*p* < 0,05) (Fig. 6c–d). In the D + N-CM group, there was no statistically significant change in both ACR (654 ± 63 ug/mg and 716 ± 35 ug/mg) and CCR (0,90 ± 0,12 ml/min and 0,95 ± 0,06 ml/min) between pre- and post-treatment periods. In the D + DFS-CM group, ACR (782 ± 70 ug/mg and 666 ± 30 ug/mg) decreased and CCR (0,83 ± 0,08 ml/min vs. 0,89 ± 0,07 ml/min) increased, but those changes were not statistically significant (Fig. 6c–d).

The percentages of weight change from pre- to post-treatment were compared among the groups; while there was weight gain in the control group, there was significant weight loss in the experimental groups. At this point, the weight loss in the D group (− 23 ± 6%) was statistically higher than the ones of D + N-CM (− 3 ± 3%) and D + DFS-CM (− 6 ± 2%) groups (Fig. 6e). Finally, the renal mass indices (RMIs) were compared among the groups, and it was noted that the RMIs of the experimental groups increased significantly compared to the C group (0,34 ± 0,01) (*p* < 0,001). When the experimental groups were compared among themselves, there was a decrease in both D + N-CM (0,63 ± 0,04) and D + DFS-CM (0,58 ± 0,01) compared to D group (0,69 ± 0,03), but the decrease was only statistically significant for the D + DFS-CM group (*p* < 0,05) (Fig. 6f).

### Light microscopic analysis

#### H&E

**Fig. 7:**
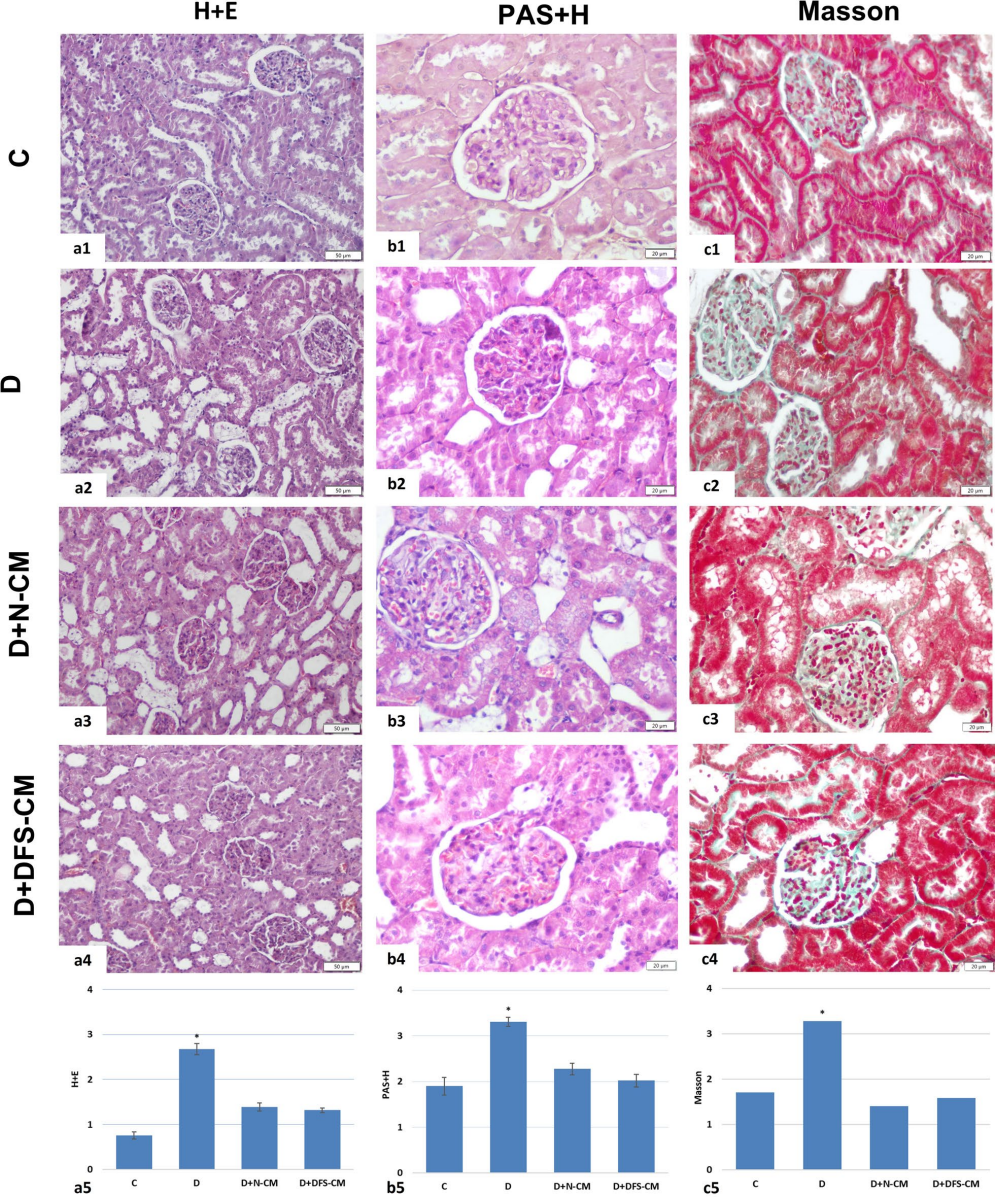
Representative micrographs of H&E (a1-4), PAS&H (b1-4) and Masson’s trichrome (c1-4) staining. General evaluation including histomorphological changes in tubular and interstitial areas was made with H&E staining (a1-4). Expansion in mesangial area enlargement and increase in GBM thickness were detected by PAS&H staining (b1-4). The development of glomerular fibrosis was demonstrated by Masson's triple staining (c1-4). Graphical representations of semi-quantitative evaluations after H&E, PAS&H and Masson’s triple staining (a5, b5, c5, respectively). *P < 0,001 vs the other groups

The groups were semiquantitatively evaluated by scoring from 0 (less than 5%) to + 3 (over 75%) in accordance with the percentage of areas with tubular damage defined with vacuolar degeneration and pyknotic nuclei, enlargement of interstitium and presence of cellular debris in the tubule lumens (Fig. 7a). There was a significant increase in the score of the D group compared to the C group. A significant decrease was observed in both treatment groups (D + N-CM and D + DFS-CM) compared to the D group (*p* < 0,001) (Fig. 7a5).

#### PAS&H

The groups were semiquantitatively assessed by scoring from 0 to + 4 in terms of mesangial area expansion and increase in GBM thickness (Fig. 7b). A significant increase was detected in the D group compared to the C, and it was found to be significantly decreased in the treatment groups (D + N-CM and D + DFS-CM) compared to the D group (*p* < 0,001) (Fig. 7b5).

#### Masson’s trichrome

The groups were examined by semiquantitative scoring from 0 to + 4 for the development of glomerular fibrosis (Fig. 7c). A significant increase was found in the D group compared to the control and treatment groups (*p* < 0,001) (Fig. 7c5).

### Transmission electron microscopic (TEM) analysis

#### Glomerular area

**Fig. 8:**
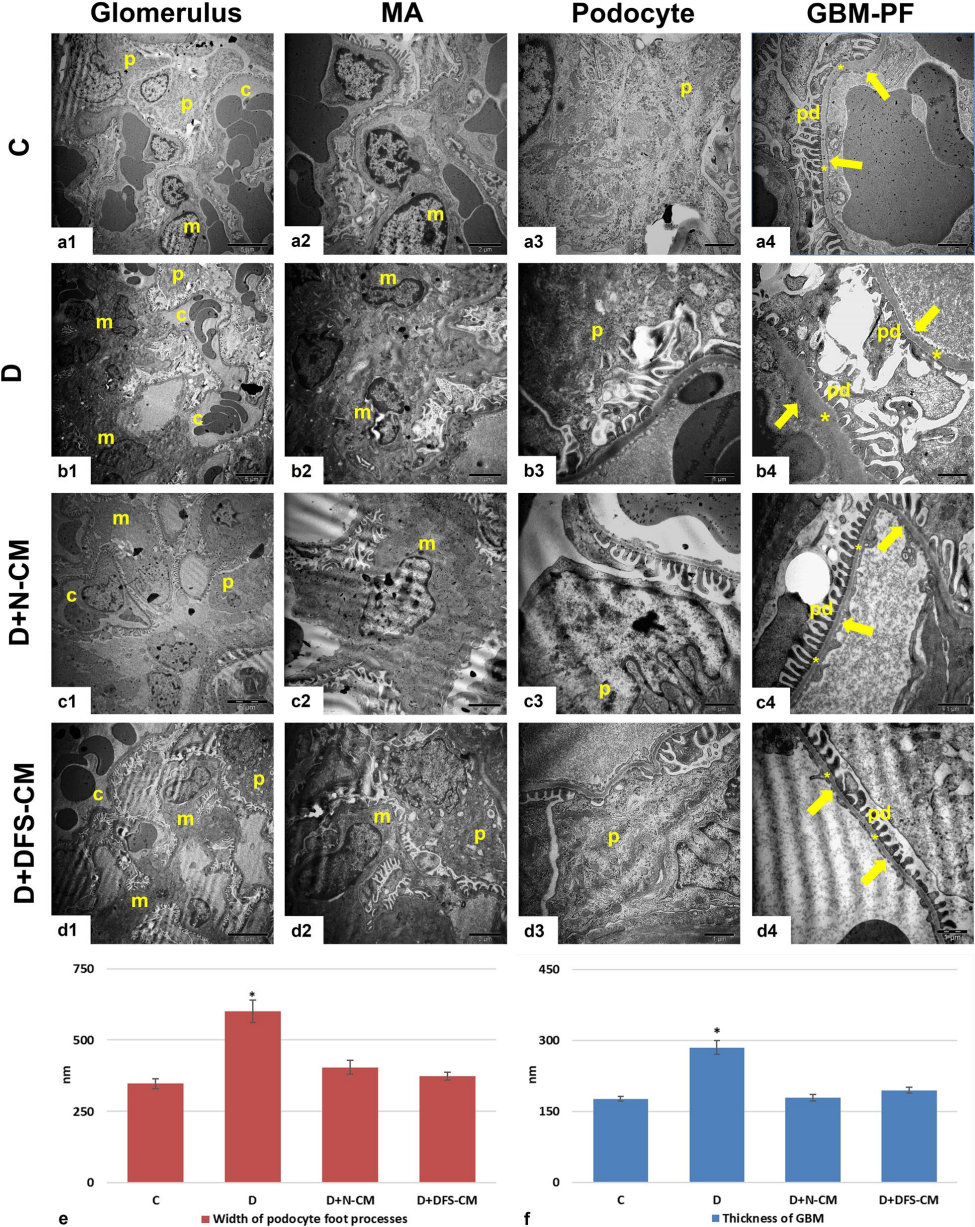
Representative TEM ultra-micrographs showing general view of glomerular area (**a1-d1**), mesangial area (**a2-d2**), podocytes (**a3-d3**), GBM and podocyte foot (PF) processes (**a4-d4**). Graphical representation of mean podocyte foot processes widths (**e**) and GBM thickness (**f**). C: capillary lumen, m: mesangial cell, p: podocyte, pd: pedicel, arrow: fenestrated endothelium of capillary, *: glomerular basal membrane *P < 0,001 vs the other groups

The glomerular areas were evaluated with TEM with respect to morphological changes specifically the presence of apoptotic body or autophagic vacuole in the mesangial cells and podocytes (Fig. 8a–d). In this respect, no apoptotic body was detected in the mesangial area or podocytes for all groups including diabetic group. Furthermore, when mesangial cells and podocytes were investigated at higher magnification, autophagic vacuoles were rarely seen (Fig. 8a–d). On the other hand, histomorphometric analysis showed that the mean width of podocyte foot processes was found to be significantly increased in the D group and there were significant decreases in both treatment groups (Fig. 8e, *p* < 0,001). Similarly, GBM thicknesses were significantly increased in diabetes group compared to the control group, while it was significantly decreased in both treatment groups compared to diabetic one (Fig. 8f, p < 0,001).

#### Cortical tubular area

**Fig. 9:**
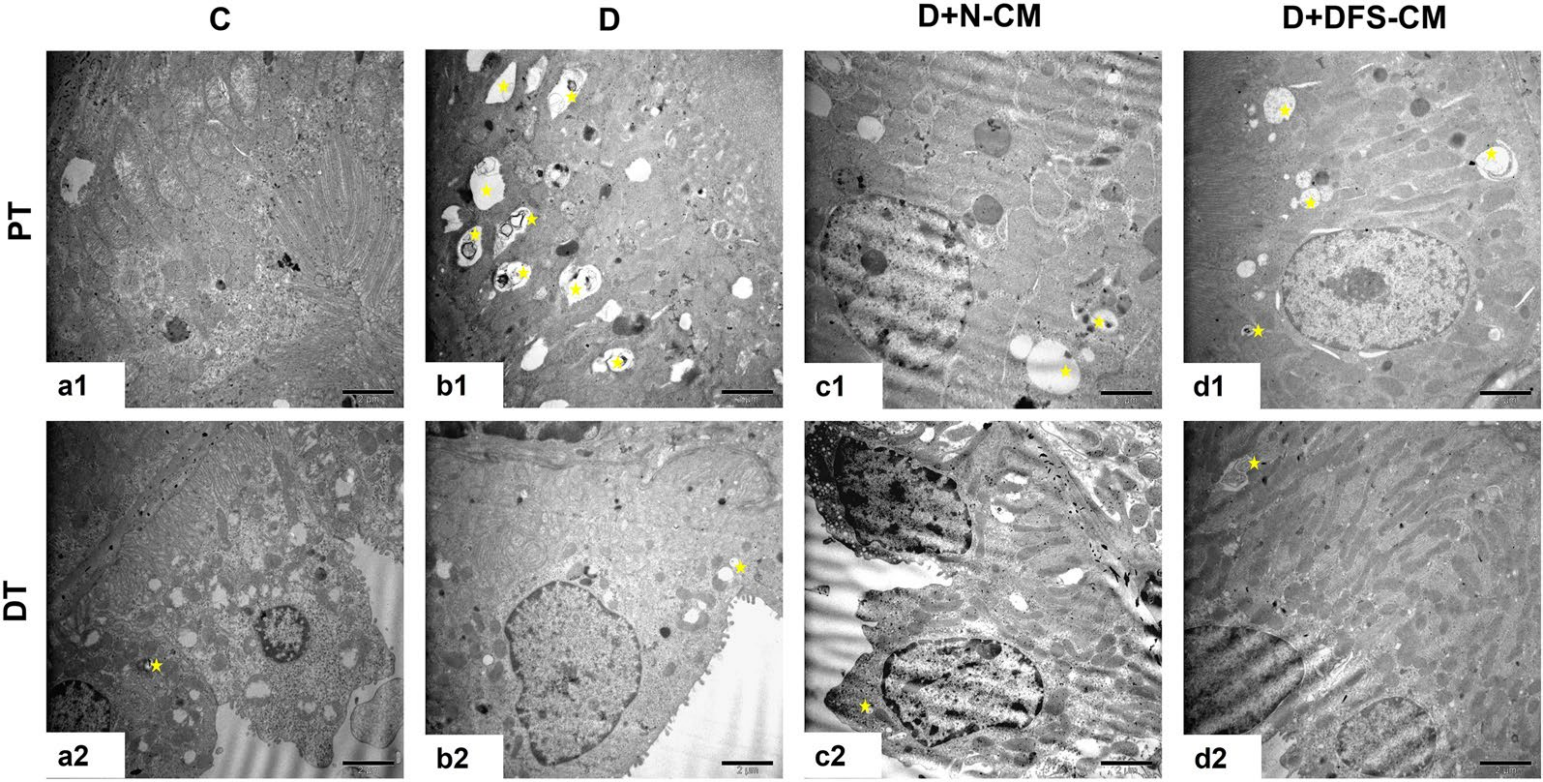
Representative TEM ultra-micrographs showing general view of proximal tubule (PT), (**a1-d1**) and distal tubule (DT), (**a2-d2**). Star: autophagic vacuole

In the diabetic group, it was observed that the amount of autophagic vacuoles in the proximal tubules was relatively more prominent than the ones in the distal tubules and the sum of autophagic vacuoles in both type of tubules was higher than one of control group. And in both treatment groups (D + N-CM and D + DFS-CM), the amount of autophagic vacuoles was on decrease compared to D group (Fig. 9).

### Immunohistochemical analysis

**Fig. 10:**
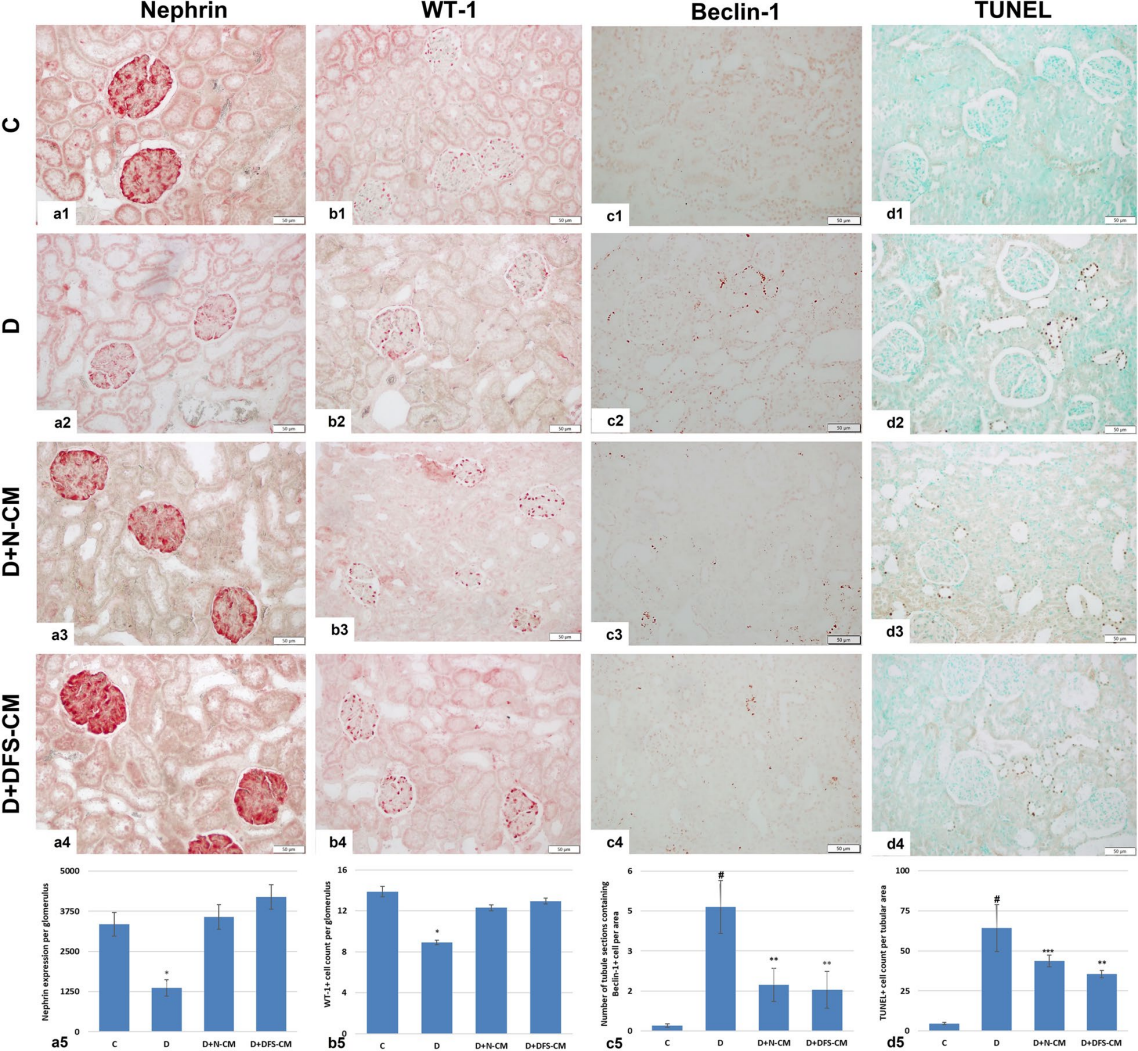
Representative micrographs of immunohistochemical staining for nephrin (**a1-4**), WT-1 (**b1-4**), beclin-1 (**c1-4**) and TUNEL (**d1-4**). Graphical representation of the results of the evaluation of immunohistochemical staining (a5, b5, c5 and d5, respectively). *P < 0,001 vs the other groups; ^#^*P* < 0,001 vs C group; ** *P* < 0,05 vs *C* and *D* groups; *** *P* < 0,05 vs *C*

*Nephrin and WT-1*: Representative micrographs of nephrin and WT-1 immunohistochemical staining are shown in Fig. 10a and Fig. 10b, respectively. While both nephrin and WT-1 expressions were significantly decreased in D group compared to C group, their expression was significantly increased in both treatment groups (D + N-CM and D + DFS-CM) (Fig. 10a5 and Fig. 10b5; *p* < 0,001).

#### Beclin-1

Cytoplasmic beclin-1 expression was not found in the glomeruli examined in all groups. However, beclin-1-positive cells were observed in the tubules and a significant increase was detected in the experimental groups compared to the control, while a significant decrease was noted in both treatment groups compared to the D group (Fig. 10c; *p* < 0,05).

#### TUNEL analysis

TUNEL-positive cells in the glomeruli examined in all groups were not sufficient for statistical evaluation. On the other hand, TUNEL-positive cells were observed in the tubules. At this point, a significant increase was detected in the experimental group compared to the control. Although there were decreases in the numbers of TUNEL-positive cells in both treatment groups, it was only statistically significant for the D + DFS-CM group compared to the D group (Fig. 10d; *p* < 0,05).

## Discussion

Mesenchymal stem cells (MSCs) have come to the forefront as an important therapeutic with their high self-renewal capacity, multipotency and low immunogenicity [46]. In addition to their regenerative potential, MSCs have been found to be therapeutically effective by their secretome and the medium containing secretome is named as conditioned medium (CM) [46]. Improving the therapeutic potential of CMs obtained from those cells would enable to increase the success of clinical applications. In this respect, preconditioning with appropriate chemical agents is one of the important strategies for the improvement of the secretome profiles of MSCs [30, 47, 48]. In this study, it has been shown that CMs obtained from hUC-MSCs after preconditioning with 150 µM DFS have higher total protein content and concentrations of growth factors including VEGF-*α*, NGF and GDNF compared to the ones of N-CM for the first time. The therapeutic effects of these two types of CMs on the rat DN model were comparatively evaluated. It was observed that both types of CMs have similar effects on biochemical, physical and histopathological analysis except for those improvements in ACR and CCR two of which were better in D + DFS-CM group than the ones in D + N-CM. In the immunohistochemical analysis, it was noted that both treatments improved the podocyte-specific nephrin and WT-1 expressions compared to D group. Tubular autophagic activity determined by beclin-1 immune-staining and electron microscopic evaluation was decreased in the treatment groups. Moreover, tubular apoptotic cell death determined by TUNEL labeling was on decrease in the treatment groups, but statistical significance was only found for the D + DFS-CM group.

DN is characterized by glomerular hyperfiltration, hypertrophy, microalbuminuria, GBM thickening and mesangial expansion in the early stages. The later stage of DN is defined by pathologies such as decreased glomerular filtration rate, macroalbuminuria, decreased creatinine clearance rate and development of fibrosis in the glomerular and tubulointerstitial areas [3, 4]. The treatments of DN which are based on lowering blood pressure and glycemic control are not fully effective in preventing the progression of the disease [19, 20]. For this reason, the development of unique treatment method that will prevent the progression of DN is the main target of studies in this field. At this point, it has been demonstrated that the capacity of MSCs to secrete therapeutically important growth factors could be increased by strategies like gene modification or preconditioning with appropriate chemical agents [28, 49]. It was shown that preconditioning of adipose tissue-derived MSCs with DFS leads to increased expression of HIF-1α and growth factors including NGF, GDNF, BDNF, VEGF-α at mRNA level [30]. NGF is important for the fully differentiation of human podocyte cells and neutralization of it results as effacement of podocyte foot processes [35]. GDNF has ameliorating effects on hyperglycemia-induced diabetic kidney damage by increasing apoptotic resistance [37]. Expression of BDNF is reduced with hyperglycemic insult which leads to disorganized actin polymerization in podocytes [36, 50]. VEGF-α has been shown to be necessary for the preservation of capillary endothelial structure and function. Furthermore, detection of proteinuria and hypertension in oncology patients using anti-VEGF reveals the necessity of VEGF-α for glomerular function [12, 51]. When the high profile of similarity between neuron and podocyte, accordingly the protective and ameliorating effects of neuro-protective and pro-angiogenic growth factors on podocyte and overall kidney injury were considered, it was decided to use DFS as a preconditioning agent to modify secretome profile of CM obtained from MSC for the sake of more effective therapeutic results. In our study, as it was expected, HIF-1α expression of MSCs preconditioned with DFS was increased compared to the ones incubated in normal conditions. The total protein concentration of DFS-CM was higher than the one of N-CM. The expressions of pro-angiogenic VEGF-α and neuro-protective NGF and GDNF in DFS-CM were also increased, while BDNF was decreased compared to N-CM. Oses et al. found out that the expressions of neuro-protective factors including NGF, BDNF and GDNF were increased at mRNA level with DFS preconditioning [30]. Therefore, our study showed that the increase in mRNA level with preconditioning with DFS was reflected at protein level in the secretome profile except for BDNF. However it must be taken account that Oses et al. used adipose tissue as a source and there is no sufficient study related to content of CMs obtained from different sources of MSC [30].

In our study, at the end of the 4th week following the single dose of 55 mg/kg STZ injection, it was determined that ACRs of the diabetic rats increased 10 times, while the CCRs decreased by 50% compared to ones of control group. These findings demonstrated that DN model was successfully induced in accordance with the criteria set by the nephropathy subcommittee of The Animal Models of Diabetic Complications Consortium [29, 52]. When the experiments were terminated, it was found out that the decrease in ACR was only statistically significant for D + DFS-CM group, while CCRs of both treatment groups were significantly improved compared to D group. Those results can be interpreted as while glomerular filtration rate was significantly ameliorated after application of both types of CMs, reduction in albumin excretion was only significant for D + DFS-CM group. The percent weight losses were significantly reduced in both treatment group, while RMI was only improved for the D + DFS-CM group compared to D group. Finally, when the pre- and post-treatment ACR and CCR values were compared, while both parameters were significantly deteriorated for D group, there was no significant change in both treatment group even if there was improvement in the D + DFS-CM group. Those results showed that applications of both types of CMs are able to stop the progression of DN. All those biochemical and physical analysis reveal that CMs obtained from MSCs under two different conditions have a similar level of therapeutic effect as direct application of MSCs [53]. On the other hand, DFS-CM seems to be more effective than N-CM with respect to their significant effects on reducing ACR and RMI.

In the light microscopic evaluations, significant reduction in tubular damage, interstitial enlargement, mesangial area expansion and development of glomerular fibrosis in both treatment group were observed compared to D group of which histopathological deterioration in the glomerular and tubular areas were generally compatible with the DN model [52, 53]. Similarly, the TEM analysis showed that GBM thickness which is on increase with DN was found to be significantly reduced in both treatment groups. Those findings indicate that both types of CMs have histomorphologically similar level of therapeutic effects.

Podocytes have primary, secondary and tertiary foot processes which interdigitates to form filtration slits and slit diaphragms connecting those tertiary foot processes have an important role in the selective permeability of glomerular filtration barrier [54]. In this respect, nephrin protein has a pivotal role as being a crucial structural component of slit diaphragm and involving in the regulation of many signaling pathways [54]. Furthermore, the decrease or mutation in its expression can often lead to severe proteinuria and lead to kidney failure [55]. In this study, it was determined that nephrin expression in D group was significantly decreased compared to C group and increased significantly in both treatment groups. A series of events including loss of nephrin expression leads to retraction or effacement of podocyte foot processes (pedicels) [56]. In this respect, the effacements of pedicels were significantly increased in D group which was compensated in both treatment groups. WT-1 protein which is another important podocyte marker has been shown to have a crucial role in the proliferation and migration of potential podocyte progenitor cells in the case of podocyte loss [57, 58]. In our study, it was found out that WT-1 expression in D group was decreased compared to the control, and then it was increased in both treatment groups. Although there is no statistical difference, the expression levels of both nephrin and WT-1 proteins in the D + DFS-CM group were higher than the ones of D + N-CM group. The decreased expressions and secretions of growth factors such as VEGF-α, NGF and GDNF play an important role in the pathogenesis of DN. Therefore, the improvements in biochemical parameters and expressions of podocyte-related proteins could be associated with increased content of VEGF-*α*, NGF and GDNF factors in the CM after preconditioning with DFS. Moreover, these finding also supports the view of that the therapeutic effects of MSCs are mediated by paracrine factors.

There are controversial findings related to the role of autophagic activity in the pathogenesis of DN. The activation status of autophagic activity in glomerular and tubular areas has been found to be inconsistent in different DN and kidney injury models [6, 17, 18]. Autophagic activation is thought to be part of the adaptation to new environmental conditions, especially in the case of cellular stress [59]. Autophagosome formation begins with the induction of proteins such as beclin-1, LC3 and Atg [59]. In our study, expression levels of beclin-1 in glomerular and cortical tubular areas were evaluated by immunohistochemical staining. Firstly, cytoplasmic beclin-1 expression was not detected in the glomerular areas of all groups. On the other hand, a significant increase was detected in the tubular area of D group compared to the C group and beclin-1 expression was significantly decreased in both treatment group. Amount of number of autophagic vacuoles was generally evaluated by TEM analysis which has been considered as gold standard for the determination of autophagic activation. TEM analyses were found to be correlated with the immunohistochemical analysis. Furthermore, the amount of autophagic vacuoles in proximal tubules was distinctively higher than the ones in distal tubules. These findings indicate that in the case of DN, the autophagic activity in the tubular area increases and therapeutic intervention like CMs obtained from MSCs reduces this autophagic activation. Proximal tubules are the main reabsorption segment of nephron which render them vulnerable to toxic factors such as albumin, high glucose and AGEs in the glomerular filtrate so that they are the most prone segment to develop tubular damage. At this point, Sakai et al. found out that autophagic activity increased in the proximal tubules, while there was no change in the distal ones in the type I mouse diabetes model induced by STZ [15, 16]. It has been suggested that the increase in the autophagic activity in the renal tubules is the kind of activation of survival mechanism against stress conditions like hyperglycemia [18]. In this respect, the decrease in autophagic activity in the treatment groups can be interpreted as the applied CMs reduce the stress factors responsible for the increase of autophagic activity in the tubules specifically proximal ones.

Persistent hyperglycemia induces the production of reactive oxygen species (ROS). Accumulation of ROS in renal parenchymal cells causes apoptosis and progression of DN [53, 60]. In this respect, there are very rare apoptotic cells in the glomeruli for both control and experimental groups. Similarly, Zhang et al. showed the presence of TUNEL-labeled apoptotic cells in the epithelial cells of tubule but did not detect them in the glomeruli [61]. The absence of apoptotic cells in the glomeruli indicates that specifically the loss of podocytes takes place through different mechanisms rather than apoptosis. The fact that podocytes with apoptotic bodies on GBM were not detected in a study in which 40,000 electron microscopic images were evaluated supports this view [62]. Therefore, podocyte loss is thought to be resulted from the activation of mechanisms such as mitotic catastrophe, epithelial–mesenchymal transformation and the process of podocyte loss triggering more podocyte loss [63, 64]. In our study, apoptotic cells in the tubules of D group were found to be significantly increased compared to control group and it was decreased in both treatment groups, but statistical significance was noted only for the D + DFS-CM group. In this respect, some studies have claimed that diabetic kidney damage primarily occurs in the tubule, especially in the proximal ones rather than in glomeruli [65]. Accordingly, this view can be supported with our results with respect to changes in the apoptotic cell death and autophagic activity status of tubular epithelial cells.

## Conclusions

Although it has been statistically demonstrated that DFS-CM application is more effective in improving parameters such as ACR and RMI, the amelioration in histopathological and immunohistochemical parameters for D + DFS-CM did not statistically differ from the ones of D + N-CM group. At this point, evaluation of the contents and therapeutic effects of CMs collected after preconditioning MSCs with higher concentrations of DFS could be important in terms of revealing the statistical difference between the two treatment groups in all parameters. Furthermore, the results of this study are worthy of note for not only demonstrating the therapeutic potential of CMs obtained from MSCs in DN but also showing that the secretome profiles of MSCs can be modified according to therapeutic targets with appropriate preconditioning strategies. In line with those results, it can be concluded that comprehensive analysis of changes in the content of CMs depending on the dose and duration of different preconditioning stimuli is of great importance for clinical applications and must be supported by in vitro and in vivo studies.

## Supplementary Information

**Additional file 1: S1:** Sample preparation for light microscopy. **S2:** Sample preparation for transmission electron microscopy.

**Additional file 2: Fig. S1: **Quantification of DFS contents in the CMs after concentration **a**, 1st **b** and 2nd **c** washes by high-performance liquid chromatography analysis.

## Data Availability

The datasets used and/or analyzed during the current study are available from the corresponding author on reasonable request.

## Abbreviations

MSC: Mesenchymal stem cell
CM: Conditioned medium
DN: Diabetic nephropathy
Huc: Human umbilical cord
DM: Diabetes mellitus
HIF-1*α*: Hypoxia-inducible factor-1 alpha
VEGF: Vascular endothelial growth factor
NGF: Nerve growth factor
GDNF: Glial-derived neurotrophic factor
BDNF: Brain-derived neurotrophic factor
TUNEL: Terminal deoxynucleotidyl transferase dUTP nick end labeling
GBM: Glomerular basal membrane
AEC: 3-Amino-9-ethylcarbazole
PBS: Phosphate-buffered saline
TEM: Transmission electron microscopy
ACR: Albumin/creatinine ratio
CCR: Creatinine clearance rate
RMI: Renal mass index
CD: Cluster of differentiation
AGEs: Advanced glycation end products
ROS: Reactive oxygen species
