# Supplementary material for: Therapeutic potential of conditioned medium obtained from deferoxamine preconditioned umbilical cord mesenchymal stem cells on diabetic nephropathy model
Source: Stem Cell Res Ther. 2022 Sep 2;13:438. doi: 10.1186/s13287-022-03121-6 (PMC9438289; doi:10.1186/s13287-022-03121-6)
Supplement: Supplementary file 1 — Additional file 1: S1: Sample preparation for light microscopy. S2: Sample preparation for transmission electron microscopy. [file 13287_2022_3121_MOESM1_ESM.docx]

**Additional file 1: S1: Sample preparation for light microscopy:** Kidney samples were placed in 10% neutral buffered formalin solution for the light microscopic investigations. After 24 hours of fixation, samples were dehydrated by incubation in increasing concentration of alcohol series (70°, 90°, 96° and 100°) and then cleared in toluene. The samples were blocked in paraffin and 4 μm thick sections were cut from the blocks. The sections were deparaffinized and rehydrated by incubating in decreasing concentrations of alcohol series (100°, 96°, 90° and 70°) and placed in distilled water.

**Additional file 1: S2**: **Sample preparation for transmission electron microscopy:**  The tissue fragments were fixed in 2,5% glutaraldehyde and then washed with 0,1 M phosphate buffer. The samples were subjected to secondary fixation in 1% osmium tetroxide at +4°C for 1 hour. Following washing with PBS, the samples were kept in 1% uranyl acetate at +4°C for 1 hour and washed with PBS again. The samples were dehydrated by incubating increasing concentration of alcohol series and then incubated in pure propylene for 20 minutes (10 min x 2) and in propylene-epon mixtures [(propylene oxide: epon (1:1), propylene oxide: epon (1:3) and pure epon] for 1 hour in each one at RT. Then the samples were blocked with polymerization in epon capsule at 60°C for 18 hours. 0,5 μm of semi-thin sections were cut from the epon blocks to determine the location of the glomeruli and then ultra-thin sections were cut on copper grids. The grids were contrasted with uranyl acetate and lead citrate for 30 minutes and 10 minutes respectively and they were investigated by transmission electron microscope (TEM; Jeol, JEM-1011).
