# Supplementary figures and images for: Therapeutic potential of conditioned medium obtained from deferoxamine preconditioned umbilical cord mesenchymal stem cells on diabetic nephropathy model
Source: Stem Cell Res Ther. 2022 Sep 2;13:438. doi: 10.1186/s13287-022-03121-6 (PMC9438289; doi:10.1186/s13287-022-03121-6)

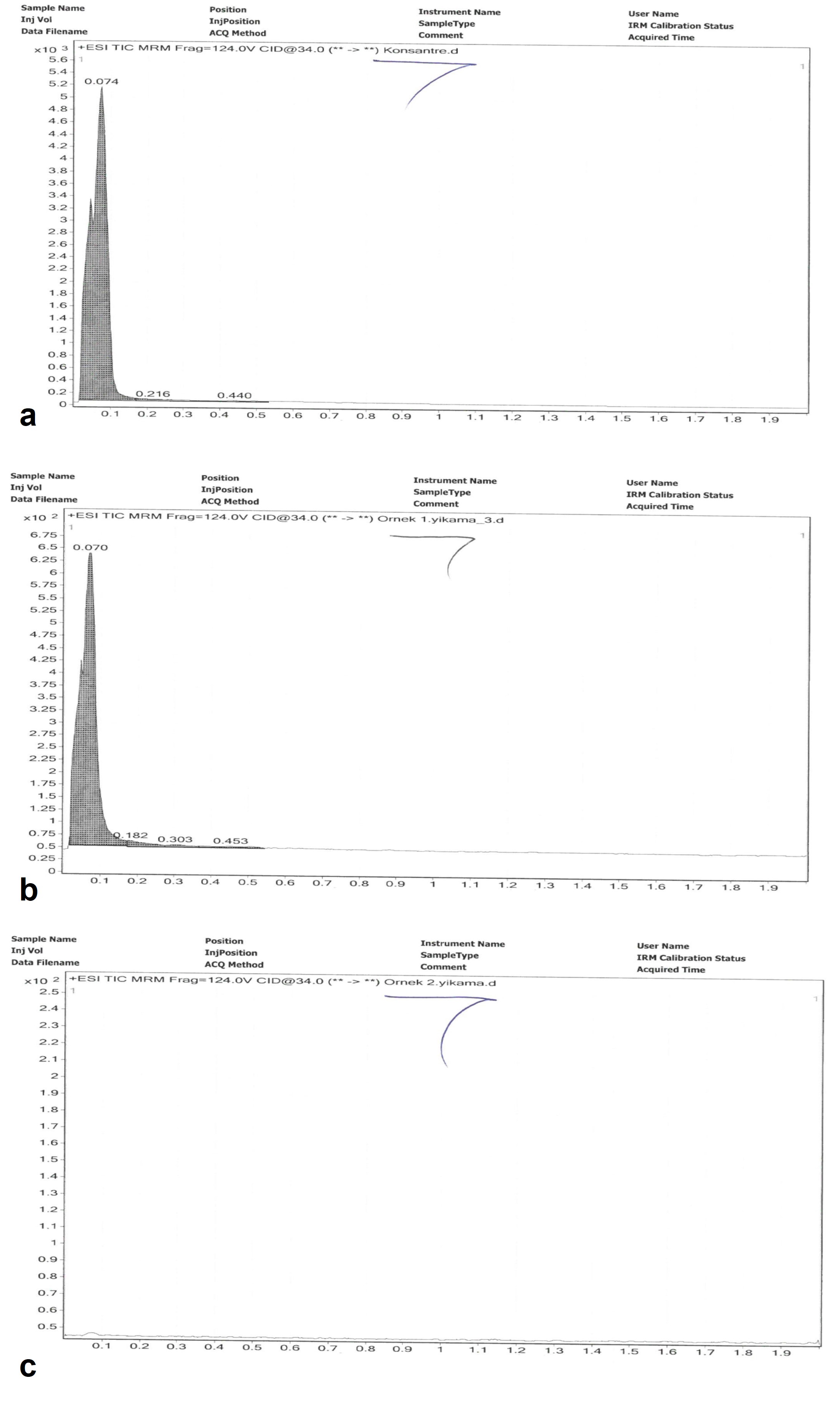

Supplement: Supplementary file 2 — Additional file 2: Fig. S1: Quantification of DFS contents in the CMs after concentration a, 1st b and 2nd c washes by high-performance liquid chromatography analysis. [file 13287_2022_3121_MOESM2_ESM.tif]
